# Mutual Coupling Reduction Between Patch Antennas Using Shorting Pin

**DOI:** 10.3390/mi17020168

**Published:** 2026-01-27

**Authors:** Junxian Li, Jiayi Zhang, Mengyan Fan, Jin Shi, Wen-Wen Yang, Lingyan Zhang, Junxiao Li, Chuan Shao, Kai Xu

**Affiliations:** 1 School of Microelectronics and School of Integrated Circuits, Nantong University, Nantong 226019, China; 18066142955@163.com (J.L.);; 2 School of Information Science and Technology, Nantong University, Nantong 226019, Chinajunxiao-li@foxmail.com (J.L.); 3 Research Center for Intelligent Information Technology, Nantong University, Nantong 226019, China; 4 Information Engineering School, Jiangsu College of Engineering and Technology, Nantong 226006, China

**Keywords:** antenna array, multiple-input multiple-output, mutual coupling reduction, patch antenna, self-decoupling, shorting pin

## Abstract

A simple self-decoupling approach using only a shorting pin is proposed to effectively reduce mutual coupling in multiple-input multiple-output patch antennas. By loading a shorting pin along the polarization direction on one side of the patch antenna, the equivalent inductance of the corresponding source is altered, thereby changing the initial phase of the slot source. This modification, in conjunction with the path effect, creates a mutual coupling null by counteracting the electric fields at the adjacent patch’s feeding position, achieving a reduced mutual coupling level. The simplicity of this decoupling method enables flexibility in practical applications, facilitating adaptation to diverse packaging environments and substrates. Furthermore, the proposed method effectively suppresses mutual coupling between adjacent and non-adjacent elements in multi-element linear arrays, as well as between elements arranged along E-planes and H-planes in planar arrays. To validate the effectiveness of this self-decoupling technique, a two-element decoupled antenna was fabricated and measured. Experimental results demonstrate a decrease in mutual coupling from −22 dB to below −40 dB across the effective frequency range of 4.809 GHz to 4.984 GHz.

## 1. Introduction

In wireless communication systems, multiple-input multiple-output (MIMO) technology has gained popularity due to its effective enhancement of channel capacity without the need for an additional spectrum [1,2]. However, the compact arrangement of elements in MIMO configurations may lead to significant electromagnetic (EM) coupling, thereby compromising the overall performance of the MIMO system [3,4]. Consequently, the development of an efficient decoupling method suitable for finite space becomes crucial.

Patch antenna arrays are a common configuration in many MIMO systems, favored for their low profile, ease of integration, and cost-effectiveness. Consequently, several methods have been applied to mitigate mutual coupling among patch antenna elements [5,6,7,8,9,10,11,12,13]. In general, these methods fall into three categories based on the locations of the decoupling structures: those situated above the antenna, those altering the ground, and those occupying the antenna or feeding layer. The first category includes methods where an array-antenna decoupling surface [5,6], metasurface superstrate [7,8,9], or high-permittivity dielectric block [10,11] is installed above the antenna. These structures effectively control the propagation of electromagnetic waves. The second category involves altering the metal ground to disrupt the field and induced currents between elements in the antenna array. Implementation forms include defected ground structure (DGS) [11,12,13,14] and decoupling ground [15]. The third category encompasses methods achieved by occupying the antenna or feeding layer, which can disrupt undesirable near-field coupling or provide an additional coupling path to counteract the initially strong coupling. Examples include the use of periodic structures [16,17,18], wave trap structures [19,20], band-stop coupled-line [21,22], half-wavelength resonator structures [23,24,25], decoupling networks [26,27,28,29], and lumped inductors [30], among others. The decoupling networks [26,27,28,29] have a minimal impact on the radiation performance of the antennas, but they can only reduce the coupling between adjacent antenna elements and are not suitable for the combination of multi-dimensional arrays. In [30], decoupling is achieved by introducing a lumped inductance between two antenna elements, allowing adjacent antennas to be placed very close to each other. However, this method results in a fixed spacing for the antenna array, making it unsuitable for antenna arrays with varying spacings.

Although the aforementioned methods demonstrate commendable decoupling performance, the introduction of parasitic structures often leads to a complex array structure, increased size, or diminished integration capability. In addressing the drawbacks associated with additional decoupling structures, new self-decoupling methods have been proposed in [31,32,33]. In [31], a weak EM interference between elements is achieved by introducing an inset-fed structure, creating a weak-field area and placing adjacent elements within this zone. This method has proven effective in decoupling two-element antenna arrays and adjacent elements in multi-element antennas. In [32], a specific null-field region for mutual coupling reduction near the feeding position of the adjacent element is generated by guiding the generation of higher-order modes and simultaneously controlling the excitation of two modes. This self-decoupling method has successfully been applied to two-element antenna arrays arranged in an E-plane or H-plane. In [33], both self-decoupling and filtering properties are achieved by integrating an inverted-F isolator into a conventional patch antenna and placing two hybrid-mode elements face-to-face to enhance the isolator’s effectiveness. Despite the effectiveness of these proposed self-decoupling schemes, it is noteworthy that in [31,32,33], little attention was given to the mutual coupling between non-adjacent elements in multi-element linear arrays and the mutual coupling in planar arrays, which remains a significant issue. In recent years, shorting-pin-loaded technology [34,35] has been used to increase the decoupling level between antenna elements.

In this article, we present a novel self-decoupling method using shorting pins to alleviate mutual coupling among patch antenna elements. Signals generated by both sides of the patch can effectively cancel each other out after traversing the two coupling paths by loading the shorting pin and meticulously adjusting various antenna parameters. This comprehensive adjustment results in the creation of a distinct null-field at the feeding position of the coupled port, leading to a notable suppression of mutual coupling. The proposed approach demonstrates its efficacy in suppressing mutual coupling among antenna elements positioned at different center-to-center distances or arranged differently, while maintaining adaptability to varying substrates. Leveraging these universal characteristics, the self-decoupling approach can be extended to multi-element linear arrays and planar arrays, with all schemes thoroughly validated. The antenna decoupling mechanism is illustrated by an equivalent circuit, a sketch of the self-decoupled concept, antenna comparisons, field analysis, and a parametric study. The design procedure of the proposed self-decoupled patch antennas is summarized to guide practical application. The performance comparisons between this work and other designs are also demonstrated.

## 2. Proposed Decoupling Technique

### 2.1. Antenna Configuration

**Figure 1:**
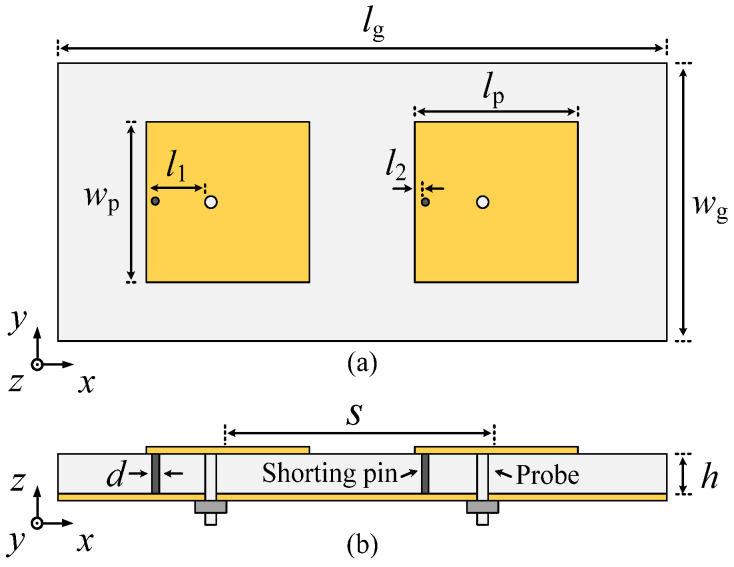
Configuration of the proposed two-element self-decoupled patch antenna using shorting pins. (**a**) Top view. (**b**) Side view.

Figure 1 illustrates the configuration of the proposed two-element self-decoupled patch antenna array. Each antenna element comprises a rectangular patch, a shorting pin, and a probe. Two antenna elements are positioned side by side along the *x*-axis, with their center-to-center distance denoted by *s*. Each element has a shorting pin inserted on the same side to achieve mutual coupling reduction. The antenna element is fed by a probe, with both the feeding point and the shorting pin located along the centerline in the *y*-axis direction.

The proposed two-element self-decoupled patch antenna array can be fabricated on a single-layer RO4003C substrate with a dielectric permittivity of 3.38, a loss tangent of 0.0027, and a thickness of *h* = 2.437 mm. Full-wave simulation is conducted using Computer Simulation Technology (CST).

### 2.2. Antenna Decoupling Mechanism

**Figure 2:**
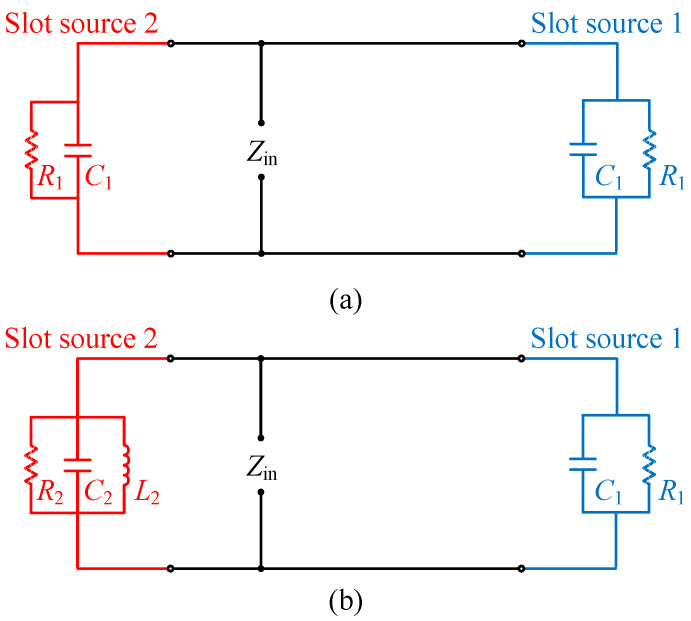
Simplified equivalent circuit model of the proposed self-decoupled patch antenna element without and with shorting pin. (**a**) Without. (**b**) With.

To further illustrate the self-decoupling mechanism, Figure 2 depicts the simplified equivalent circuit model of the proposed patch antenna element without and with a shorting pin. As is widely known, a rectangular microstrip antenna can be represented as an array of two radiating narrow apertures (slots) [36]. For the proposed antenna, the radiating slot named Slot source 1 on the side without a shorting pin can be modeled as a parallel capacitance (*C*_1_) and resistance (*R*_1_), similar to a conventional patch antenna. On the other side, which features a shorting pin, the equivalent circuit model of the radiating slot named Slot source 2 will include an additional parallel equivalent inductance (*L*_2_), along with the equivalent capacitance (*C*_2_) and resistance (*R*_2_). Notably, *C*_2_ and *R*_2_ differ from *R*_1_ and *C*_1_ due to the unilateral loading of inductance, causing the edge field on the corresponding side to differ from the other side. Hence, a transmission null can be obtained for improving the decoupling level.

**Figure 3:**
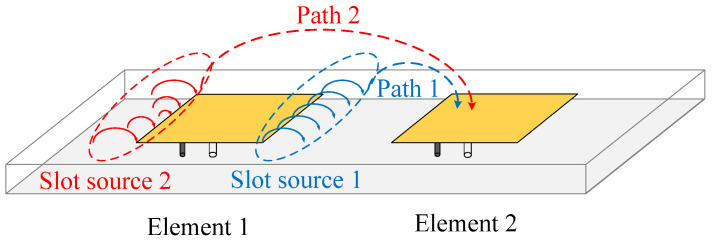
Sketch of the proposed self-decoupled concept.

Given the different circuit characteristics on the two sides of the patch antenna, signals generated from Slot sources 1 and 2 will traverse two different coupling paths, named Path 1 and Path 2, respectively, as depicted in Figure 3. The patch widths on both sides are identical, and the difference in location between the two slot sources minimally affects the magnitudes of their respective signals. Consequently, it can be assumed that the signal magnitudes of the two paths are approximately equal.

To achieve effective mutual coupling reduction, it is crucial for the signal transmitted from Element 1 to Element 2, passing through the two paths, to undergo a 180° phase difference, which is essential for path cancelation. The phase difference between Path 1 and Path 2 in a consistent transmission environment depends on two factors: the initial phase and the transmission distance. For the proposed antenna, the significant impact on the initial phase of the signal generated by the two slot sources stems from the differences in circuit characteristics illustrated in Figure 2.

The transmission distance is predominantly influenced by the electrical length of the patch (*l*_p_) in the polarization direction and the center-to-center distance between antenna elements. Furthermore, the feeding position of the patch antenna can also affect these two factors, as it influences the excitation of the slot sources, as well as the resulting scattering field and radiation characteristics of the antenna.

**Figure 4:**
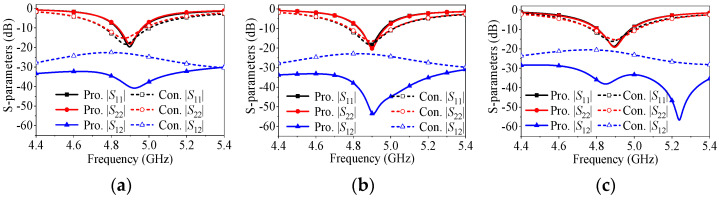
*S*-parameters of the proposed and conventional patch antenna arrays when center-to-center distance is (**a**) 0.6λ_0_, (**b**) 0.5λ_0_ or (**c**) 0.4λ_0_.

As previously analyzed, it is predictable that the proposed antenna element can achieve self-decoupling by carefully selecting key dimensions of critical parts, relying on path cancelation caused by combined radiating slot sources on both sides. To validate the effectiveness and universality of the proposed self-decoupling method, Figure 4 presents simulated *S*-parameters for three different cases, respectively, comparing the mutual coupling levels of the proposed and a conventional two-element patch antenna array at varying center-to-center distances. Both antennas operate in the TM_01_ mode with a design frequency of 4.9 GHz, a potential band for 5G Sub-6 GHz. In contrast to conventional patch antenna arrays, the proposed self-decoupled patch antenna arrays achieve mutual coupling nulls within the operating frequency range at center-to-center distances of 0.6λ_0_, 0.5λ_0_, and 0.4λ_0_, respectively. Furthermore, the mutual coupling level is reduced from about −22 dB to at least −32 dB, and the wide decoupling frequency range is maintained.

**Figure 5:**
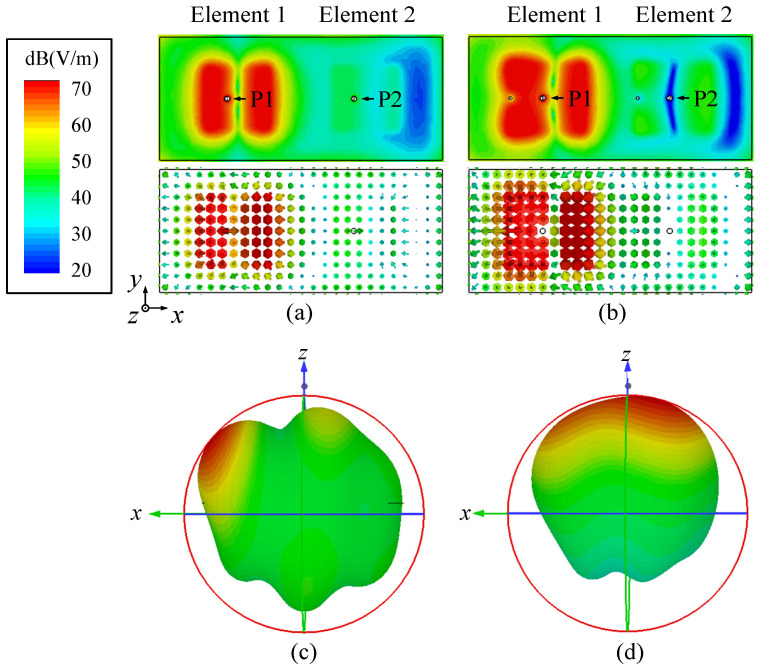
Simulated average magnitude and vector distributions of *E*-fields and radiation patters in two-element patch antenna arrays with center-to-center spacing of 0.5λ_0_ at center frequency. (**a**,**c**) Conventional patch antenna array. (**b**,**d**) Proposed self-decoupled patch antenna array in Figure 1.

Figure 5 illustrates the average magnitude and vector distributions of *E*-fields in conventional and proposed patch antenna arrays at a center-to-center distance of 0.5λ_0_, with Element 1 excited and Element 2 terminated with a 50 Ω load. In Figure 5a, for the conventional patch antenna array, the port position of the adjacent antenna element demonstrates a strong *E*-field, potentially causing poor isolation between the two ports. In Figure 5b, the proposed two-element self-decoupled antenna array exhibits a significant decrease in the *E*-field strength around the adjacent coupled element port. This outcome underscores the effective suppression of mutual coupling between elements through the implementation of this decoupling method. Furthermore, Figure 5c,d show that the radiation pattern can recover after adding a shorting pin.

### 2.3. Parametric Study

**Figure 6:**
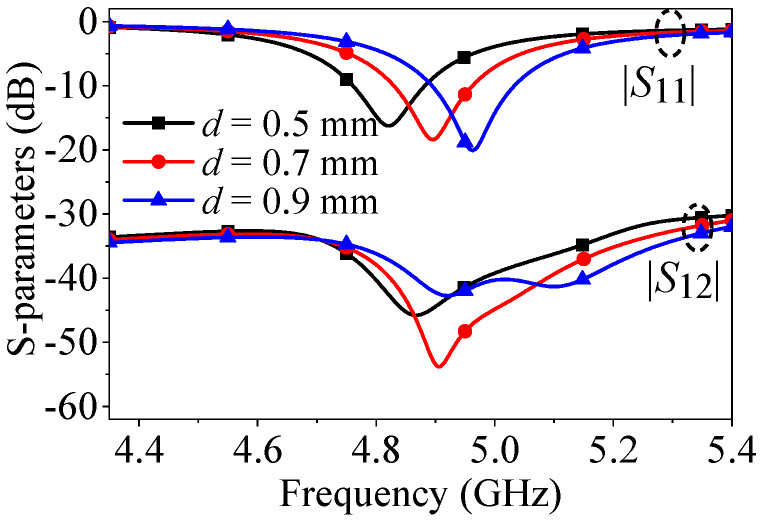
*S*-parameters of the proposed two-element patch antenna when center-to-center spacing is 0.5λ_0_ with different *d*.

In Figure 6, it is evident that as the diameter of the shorting pin (d) increases, both the reflection zero and the mutual coupling null shift to higher frequencies. This occurs because an increase in the pin diameter is equivalent to a reduction in inductance L2, as shown in Figure 2, which results in a change in the equivalent electrical length of the patch and the changed initial phase of Slot source 2. Consequently, the frequencies of both resonance and path cancelation undergo certain changes.

**Figure 7:**
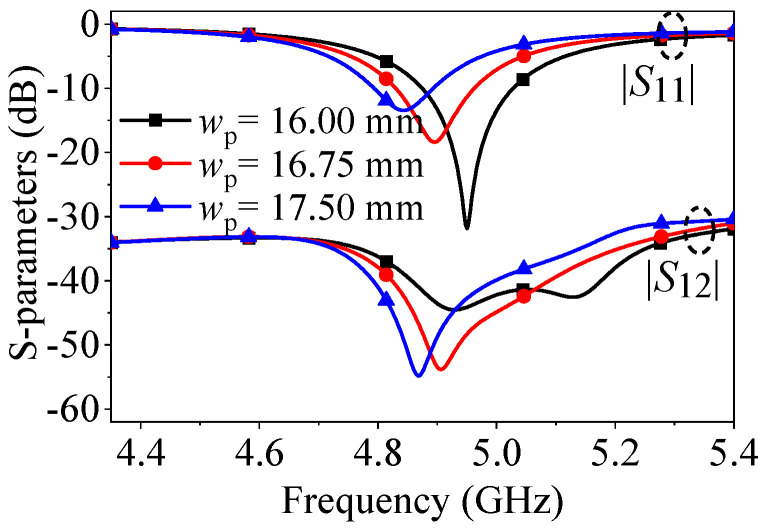
*S*-parameters of the proposed two-element patch antenna when center-to-center spacing is 0.5λ_0_ with different *w*_p_.

Figure 7 demonstrates that an increase in the width of the patch (*w*_p_) leads to the reflection zero and the mutual coupling null shifting to lower frequencies. This is due to the widening of the patch, resulting in an increase in the equivalent capacitance values *C*_1_ and *C*_2_, as shown in Figure 2. This increase in capacitance leads to an enlarged resonant cavity size (extended equivalent electrical length) and changes in the initial phases of Slot source 1 and Slot source 2. Therefore, the frequencies of both resonance and path cancelation change simultaneously.

**Figure 8:**
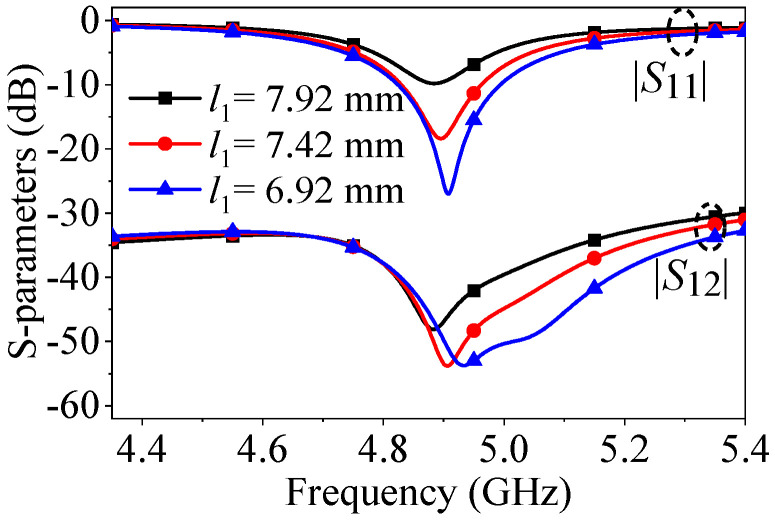
*S*-parameters of the proposed two-element patch antenna when center-to-center spacing is 0.5λ_0_ with different *l*_1_.

Figure 8 illustrates that as the feeding position (*l*_1_) changes, the impedance matching level improves because different location of the feeding will cause different input impedance. At the same time, the mutual coupling null shifts to a higher frequency due to alterations in the initial phase distributions of the two sources and the radiation characteristics of the patch. These changes cause a slight shift in the frequency of path cancelation.

**Figure 9:**
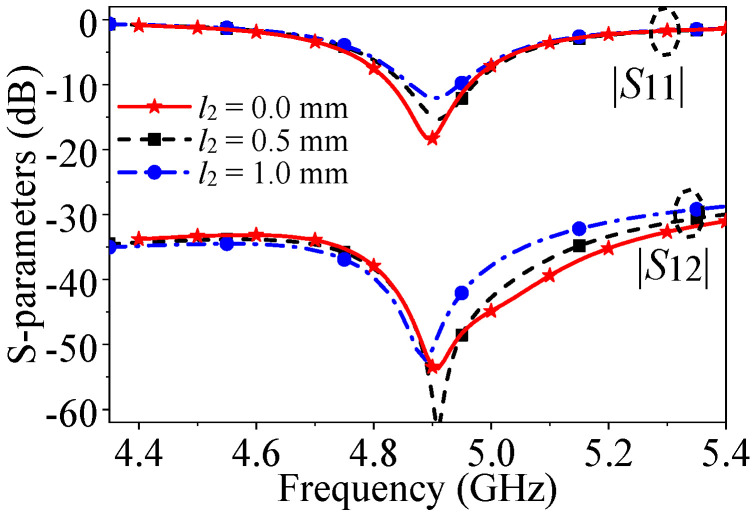
*S*-parameters of the proposed two-element patch antenna when center-to-center spacing is 0.5λ_0_ with different *l*_2_.

In Figure 9, it is evident that when the distance between the shorting pin and the edge (*l*_2_) increases, the impedance matching level slightly deteriorates. This is attributed to changes in the *E*-field distribution affecting the matching position. Additionally, the mutual coupling null shifts to a lower frequency as the position of the shorting pin affects the initial phase of the signal generated from Slot source 2, thereby modifying the frequency of path cancelation. Therefore, it can be found from Figure 6 that the changes in the shorting pin will affect the coupling level of the patch antenna, which results from the change in equivalent inductance introduced by the shorting pin.

**Figure 10:**
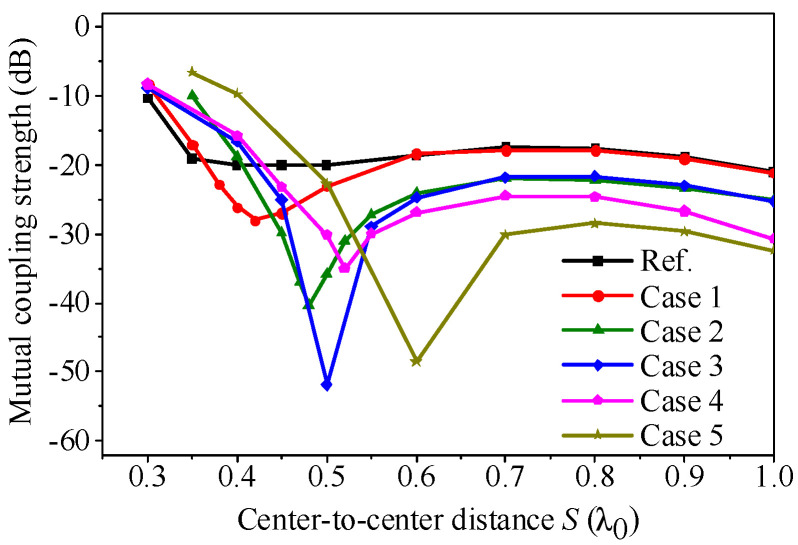
Mutual coupling strength of different cases in different center-to-center distances.

To assess the adaptability of the proposed self-decoupling approach to different substrates, a comparison of mutual coupling strength among various array cases has been conducted. The dimension parameters of these cases are listed in Table 1, all designed to operate at 4.9 GHz. The length of the ground plane (*l*_g_) in Figure 1 has been adjusted to 2.0λ_0_ to provide sufficient location space for arranging antenna elements when the array parameters vary. Figure 10 presents a comparison of mutual coupling strengths among different two-element patch antenna arrays at varying center-to-center distances (*S*). As a point of reference, the coupling strength versus spacing of the two-element conventional patch antenna array is included. The conventional array (black curve) does not exhibit a mutual coupling null in the curve at any spacing, while all the listed cases display an obvious mutual coupling null. This indicates that the proposed self-decoupling method is applicable to different substrates by adjusting the size of the patch and the location of the feeding probe.

### 2.4. Design Procedure

According to the above analysis, the design procedure for the proposed self-decoupled patch antenna can be summarized as follows:

(1) Obtain the initial dimensions of *l*_p_, *w*_p_ and *l*_1_ based on *f*_0_.

(2) Utilize the antenna decoupling mechanism, setting *l*_p_ = 0.296λ_0_, *w*_p_ = 0.247λ_0_, *l*_1_ = 0.122λ_0_, and the initial values for *d* and *l*_2_.

(3) Fine-tune the dimensions slightly based on the antenna decoupling mechanism and variations in the parameters shown in Figure 6, Figure 7, Figure 8, Figure 9 and Figure 10 to obtain the final dimensions.

## 3. Simulated and Measured Results

### 3.1. Two-Element Self-Decoupled Patch Antenna Array

**Figure 11:**
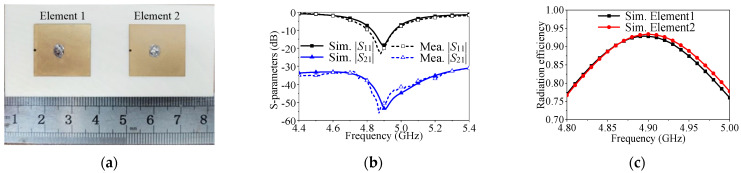
Photograph, simulated and measured S-parameters and efficiency of the proposed two-element antenna array. (**a**) Photograph. (**b**) S-parameters. (**c**) Efficiency.

To further validate the feasibility of mutual coupling suppression, a prototype of the proposed two-element self-decoupled patch antenna array is demonstrated and measured. Figure 11a presents the photograph of the prototype. According to the design procedure, the detailed dimensions of this design are as follows: *l*_g_ = 68 mm, *w*_g_ = 30 mm, *l*_p_ = 18.15 mm, *w*_p_ = 16.75 mm, *l*_1_ = 7.43 mm, *l*_2_ = 0 mm, *d* = 0.7 mm and *s* = 30.6 mm. *S*-parameters are measured using a vector network analyzer (Keysight N5230C, Keysight Technologies, Inc., Santa Rosa, CA, USA), and gain and radiation patterns are measured inside an anechoic chamber with a far-field antenna measurement system. One antenna element is excited while the other is terminated with a 50 Ω load.

The simulated and measured *S*-parameters of the proposed decoupled two-element antenna array are presented in Figure 11b. The measured 10 dB impedance matching bandwidth is 0.175 GHz (4.809–4.984 GHz), or 3.57%. The mutual coupling level is below −40 dB, with the minimum value reaching −55 dB, representing a 16 dB improvement compared to the array without decoupling.

**Figure 12:**
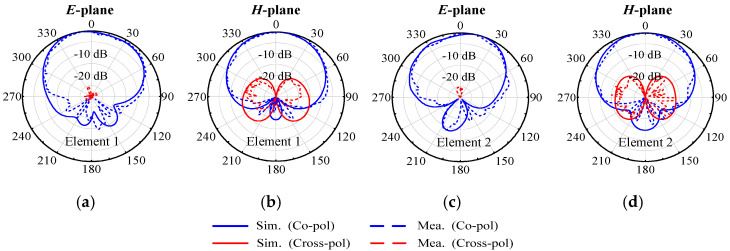
Simulated and measured radiation patterns of the proposed two-element patch antenna array at center frequency. (**a**) *E*-plane of Element 1. (**b**) *H*-plane of Element 1. (**c**) *E*-plane of Element 2. (**d**) *H*-plane of Element 2.

Figure 12 displays the measured and simulated radiation patterns of the proposed two-element antenna array at 4.9 GHz, with both elements measured separately. The cross-polarization level is below −19 dB, and there is good consistency between the simulated and measured results.

### 3.2. Four-Element Self-Decoupled Linear Patch Antenna Array

**Figure 13:**
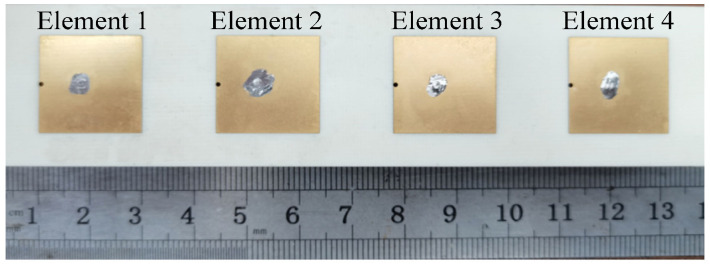
Photograph of the proposed four-element antenna array (*l*_g_ = 124.8 mm, *w*_g_ = 30 mm, *l*_p_ = 17.8 mm, *w*_p_ = 17.8 mm, *l*_1_ = 6.74 mm, *l*_2_ = 0 mm, *d* = 0.7 mm and *s* = 30.6 mm).

**Figure 14:**
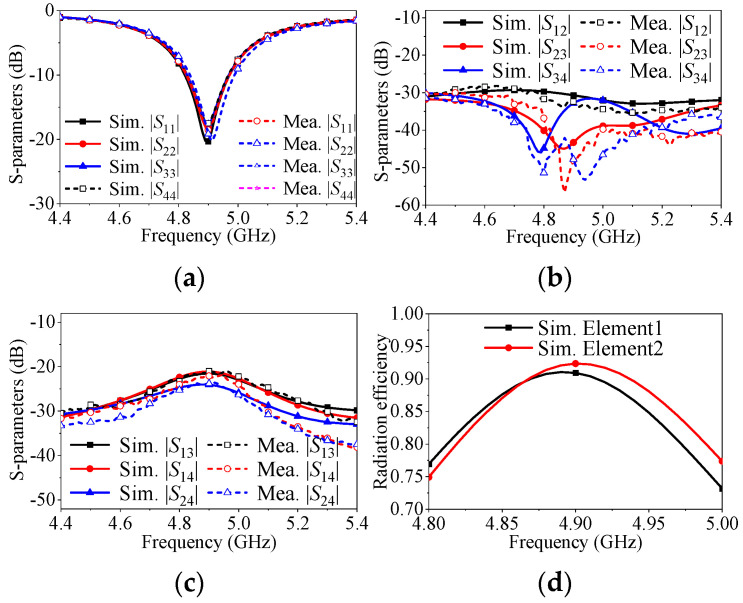
S-parameters of the proposed four-element antenna array for adjacent elements decoupling and conventional four-element antenna array when center-to-center spacing is 0.5λ_0_. (**a**) |*S*_11_|, |*S*_22_|, |*S*_33_| and |*S*_44_|. (**b**) |*S*_12_|, |*S*_23_| and |*S*_34_|. (**c**) |*S*_13_|, |*S*_14_| and |*S*_24_|. (**d**) Radiation efficiency.

To investigate the application of the self-decoupling method in a multi-element linear array, a prototype of a four-element linear array with an element spacing of 0.5λ_0_ is fabricated and measured, as depicted in Figure 13. The reflection responses are illustrated in Figure 14.

In Figure 14a, it is evident that the proposed approach has a negligible impact on impedance matching for the four elements, even with different electromagnetic environments in the array, as their reflection coefficients are nearly identical. Figure 14b,c present the coupling characteristics of the proposed four-element antenna array. The mutual coupling between any two adjacent elements (|S_12_|, |S_23_|, or |S_34_|) is below −30 dB, while the conventional patch measures only −22 dB. The mutual coupling between two non-adjacent elements (|*S*_13|_, |*S*_14_|, and |*S*_24_|) is maintained at almost the same level as the conventional antenna array, which is lower than −21 dB.

**Figure 15:**
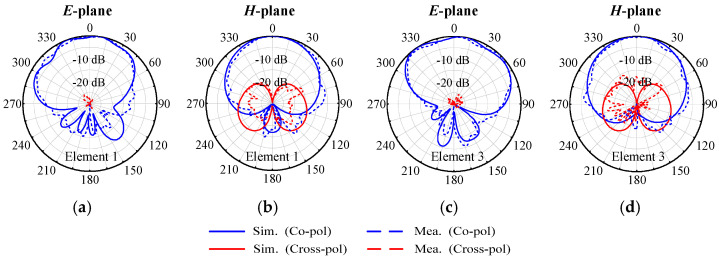
Simulated and measured radiation patterns of the proposed four-element self-decoupled linear patch antenna array at center frequency. (**a**) *E*-plane of Element 1. (**b**) *H*-plane of Element 1. (**c**) *E*-plane of Element 3. (**d**) *H*-plane of Element 3.

The measured radiation patterns of the four-element array at 4.9 GHz are depicted in Figure 15. The measured 3 dB beamwidths at 4.9 GHz of the E-plane and H-plane are from 87.6° to 112.4° and 89.0° to 89.4°, respectively. The measured maximum cross-polarization levels within the 3 dB beam range in the *E*-plane and *H*-plane are from −25.2 to −28.4 and −19.3 to −20.1 dB, respectively. These results indicate that the four-element array achieves notable radiation performance.

## 4. Discussion of Self-Decoupled Four-Element Antenna Arrays

### 4.1. Decoupling Between Non-Adjacent Elements in Four-Element Linear Patch Antenna Array

While the proposed self-decoupling approach effectively reduces mutual coupling between adjacent elements in a four-element linear array, its effectiveness is limited for non-adjacent elements due to significant changes in both initial phase and paths. This limitation can be addressed by adjusting key parameters.

**Figure 16:**
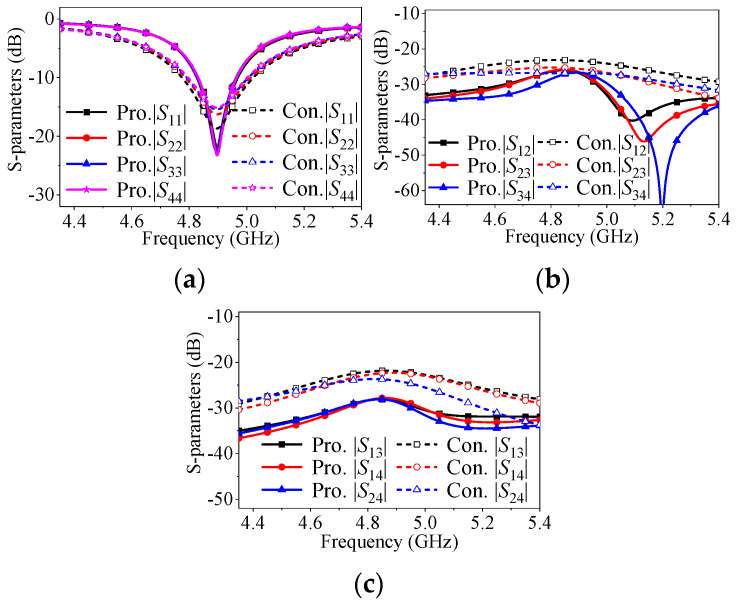
*S*-parameters of the proposed four-element antenna array for non-adjacent elements decoupling and conventional four-element antenna array when center-to-center spacing is 0.5λ_0_. (**a**) |*S*_11_|, |*S*_22_|, |*S*_33_| and |*S*_44_|. (**b**) |*S*_12_|, |*S*_23_| and |*S*_34_|. (**c**) |*S*_13_|, |*S*_14_| and |*S*_24_| (*l*_g_ = 128 mm, *w*_g_ = 30 mm, *l*_p_ = 18.15 mm, *w*_p_ = 16.75 mm, *l*_1_ = 7.43 mm, *l*_2_ = 0 mm, *d* = 0.7 mm and *s* = 30.6 mm).

Figure 16c illustrates the coupling characteristics of another four-element antenna array case, verifying the effectiveness between non-adjacent elements. It is evident that the mutual coupling level between any two non-adjacent elements, such as |*S*_13_|, |*S*_14_|, and |*S*_24_|, is significantly reduced from approximately −22 dB to below −29 dB. In contrast, the mutual coupling between two adjacent elements (|*S*_12_|, |*S*_23_|, or |*S*_34_|) remains at the same level compared to the conventional array. Therefore, for different application scenarios, the proposed approach can achieve adaptation by flexibly solving the coupling between adjacent or non-adjacent elements.

### 4.2. Four-Element Self-Decoupled Dual-Polarized Planar Array

**Figure 17:**
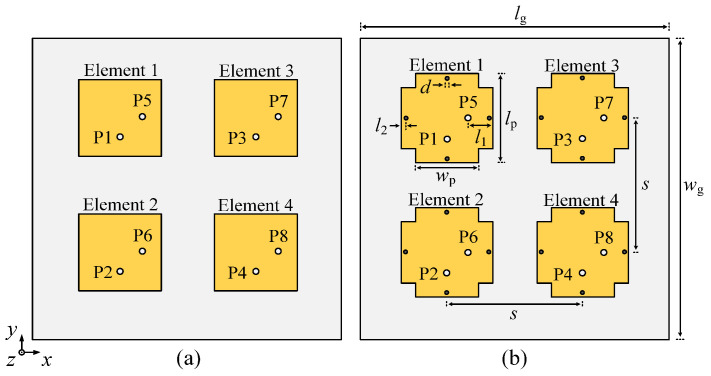
Configuration of four-element dual-polarized planar patch antenna array. (**a**) Conventional four-element planar array. (**b**) Proposed self-decoupled four-element planar array (*l*_g_ = 102 mm, *w*_g_ = 102 mm, *l*_p_ = 32 mm, *w*_p_ = 20.8 mm, *l*_1_ = 10 mm, *l*_2_ = 0 mm, *d* = 1.2 mm and *s* = 51.42 mm).

**Figure 18:**
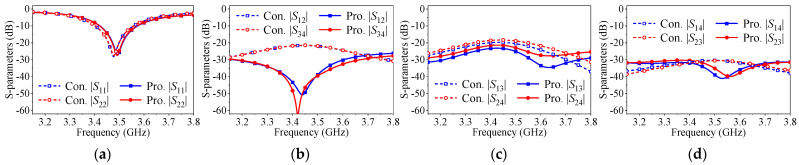
*S*-parameters of the proposed and conventional four-element dual-polarized planar patch antenna array. (**a**) |*S*_11_|, |*S*_22_|, |*S*_33_| and |*S*_44_|. (**b**) |*S*_12_| and |*S*_34_|. (**c**) |*S*_13_| and |*S*_24_|. (**d**) |*S*_14_| and |*S*_23_|.

The proposed self-decoupling method is also effective in reducing mutual coupling among elements in a planar array. Figure 17 shows the configuration of a conventional dual-polarized four-element planar array without any decoupling steps and a self-decoupled planar array integrating the proposed method. The difference between the two arrays in |*S*_11_|, |*S*_22_|, |*S*_33_| and |*S*_44_| in Figure 18a is subtle, indicating that the different EM environments in the planar array have insignificant effect on impedance matching.

Figure 18b demonstrates the coupling characteristics between elements arranged along the *E*-plane, where |*S*_12_| decreases from −20 dB to −25 dB, and |*S*_34_| decreases from −19 dB to −24 dB. Figure 18c shows the coupling characteristics between elements arranged along the H-plane, where |*S*_13_|, |S_24_| all decrease from −23 dB to −32 dB. Similar conclusions apply to |*S*_57_| and |*S*_68_| because of the symmetrical structure. Figure 18d shows the coupling characteristics between elements arranged along the diagonal, with both |*S*_14_| and |*S*_23_| decreasing from −27 dB to −30 dB.

The proposed approach is capable of achieving mutual coupling reduction for elements arranged along the *E*-plane and *H*-plane in a planar array simultaneously. It is worth noting that the center-to-center spacing of elements arranged along the diagonal is relatively wide, so the mutual coupling in the conventional four-element planar array is relatively weak, making the improvement less noticeable.

**Figure 19:**
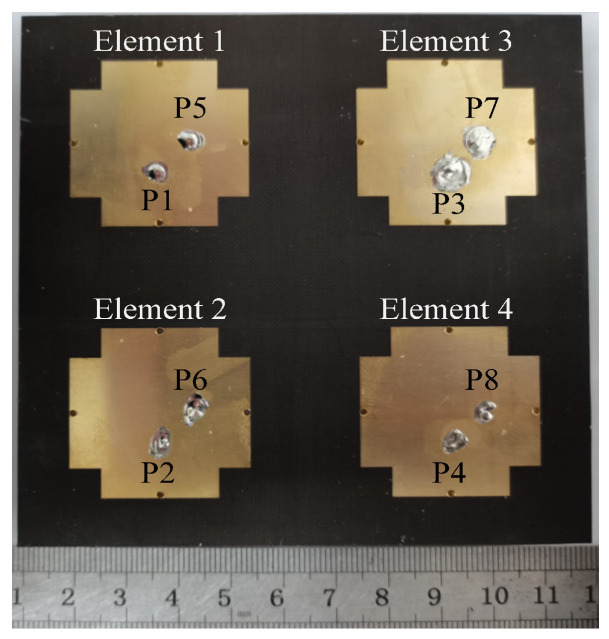
Fabricated prototype of the proposed four-element self-decoupled dual-polarized planar patch antenna array.

**Figure 20:**
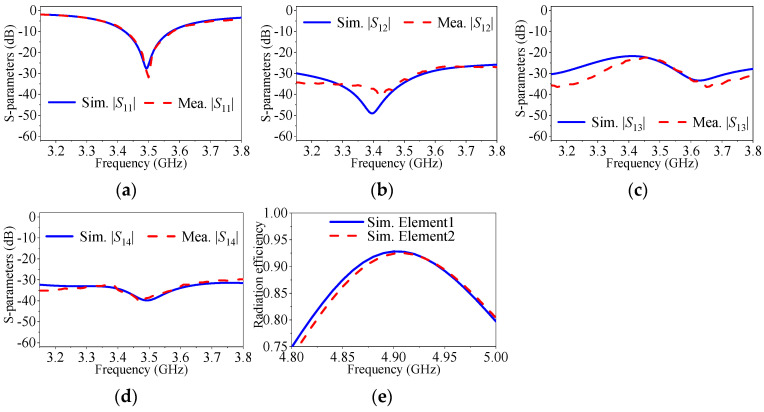
*S*-parameters of the proposed and conventional four-element dual-polarized planar antenna array. (**a**) |*S*_11_| (|*S*_22_|, |*S*_33_| and |*S*_44_|). (**b**) |*S*_12_|. (**c**) |*S*_13_|. (**d**) |*S*_14_|. (**e**) Efficiency.

Figure 19 displays a photo of the fabricated four-element self-decoupled dual-polarized planar patch antenna array, and Figure 20 exhibits a comparison of its simulated and measured S-parameters. The measured responses in Figure 20 show satisfactory consistency with simulation results.

**Figure 21:**
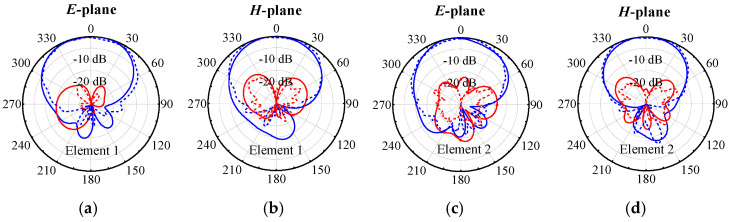
Simulated and measured radiation patterns of the proposed two-element array at center frequency. (**a**) *E*-plane of Element 1. (**b**) *H*-plane of Element 1. (**c**) *E*-plane of Element 2. (**d**) *H*-plane of Element 2.

Figure 21 displays the simulated and measured radiation patterns for Elements 1 and 2 at the center frequency, under the condition of single port excitation. The *E*-plane and *H*-plane 3 dB beamwidths of Element 1 are 89.6° and 81.5°, with cross-polarization levels of −18.5 dB and −17.2 dB. Element 2 has *E*-plane and *H*-plane 3 dB beamwidths of 87.4° and 82.1°, with cross-polarization levels of −17.4 dB and −19.1 dB. These findings indicate that the proposed self-decoupling method has no negative effects on the radiation characteristics of planar arrays, demonstrating the effectiveness and applicability of the method for decoupling applications in planar arrays.

### 4.3. Comparison

Table 2 provides a performance comparison between the proposed self-decoupling method and the state-of-the-art decoupled antennas. The proposed method stands out as it does not necessitate additional structures or circuits, thereby avoiding an increase in profile, structural complexity, or extra costs. In addition, it exhibits versatility by achieving decoupling in various application scenarios. In a two-element antenna array, it offers adaptability to different center-to-center distances or substrates through comprehensive adjustments. For a four-element linear array, the method can dynamically address coupling issues among adjacent or non-adjacent elements based on specific situations. In a four-element planar array, it simultaneously reduces mutual coupling between elements arranged along the *E*-plane and *H*-plane.

## 5. Conclusions

This article introduces a simple self-decoupling method that utilizes a shorting pin to mitigate mutual coupling among patch antenna arrays. By properly loading the shorting pin on one side of the patch antenna along the polarization direction and adjusting antenna dimensions comprehensively, effective adjustment of the initial phase is achieved. Consequently, mutual coupling nulls are consistently formed by canceling the electric field at adjacent ports, whether in a two-element array, four-element linear array, or four-element planar array. With these advantages, the proposed self-decoupling method shows promise for application in MIMO antenna systems.

## Figures and Tables

**Table 1:** Different decoupled two-element patch antenna arrays.

Case	1	2	3	4	5
*ε*_r_	4.4	3.38	3.38	3.38	2.6
*h*	2.437	1.5	2.437	3.5	2.437
*l*_P_	16	18.6	18.15	17.6	20.5
*w*_P_	14.77	17.17	16.75	16.25	18.93
*d*	0.2	0.7	0.7	0.7	0.7
*l*_1_	6.35	7.65	7.425	6.65	8.1

**Table 2:** Performance Summary of The Proposed and State-of-the-art Designs.

Ref.	Array Configuration, Center Frequency (GHz) and Bandwidth (%)	Center-To-Center Distance (λ_0_)	Profile (λ_0_)	Mutual Coupling Level (Mutual Coupling Reduction Level) Between Adjacent Elements (dB)	Mutual Coupling Level (Mutual Coupling Reduction Level) Between Non-Adjacent Elements (dB)	Self-Decoupling	Design Flexibility
[5]	1 × 8: 2.45, 6.1	0.45	0.36	−20 (5)	−24 (2)	No	Low
[7]	1 × 2: 2.60, 7.7 1 × 2: 3.50, 5.7	0.36 0.48	0.14 0.19	−25 (17) −27 (12)	/	No	Low
[12]	1 × 2: 2.20, 4.5	0.42	0.03	−30 (15)	/	No	Low
[15]	1 × 8: 4.90, 4.0	0.52	0.13	−27 (9)	−33 (9)	No	Low
[16]	1 × 2: 3.07, 7.0	0.75	0.09	−30 (4)	/	No	Low
[19]	1 × 2: 3.25, 1.5	0.36	0.02	−16 (6)	/	No	Low
[23]	1 × 2: 2.44, 4.9	0.60	0.07	−29 (8)	/	No	Low
[20]	1 × 8: 4.90, 13.3 4 × 2: 4.90, 13.3	0.50 0.50	0.07 0.07	−26 (7) −26.5 (7.5)	28 (10) 23.5 (5.5)	No	High
[26]	1 × 16: 7.50, 3.3	0.50	0.09	−25 (N.A.)	24 (N.A)	No	Low
[31]	1 × 2: 3.50, 3.1 1 × 4: 3.50, 3.1	0.50 0.50	0.04 0.04	−30 (5) −27 (N.A.)	/ 29 (N.A)	Yes	High
[32]	1 × 2: 5.78, 2.9 1 × 2: 5.84, 1.9	0.41 0.50	0.04 0.04	−22 (12) * −21 (3)	/	Yes	Low
[33]	1 × 2: 4.17, 2.3	0.35	0.02	−25 (8) −26 (11)	/	Yes	Low
This work	1 × 2: 4.90, 2.9 1 × 2: 4.90, 3.6 1 × 2: 4.90, 3.6 1 × 4: 4.90, 3.0 1 × 4: 4.90, 3.6 2 × 2: 3.50, 4.2	0.60 0.50 0.40 0.50 0.50 0.60	0.04 0.04 0.04 0.04 0.04 0.05	* −36 (14) −40 (18) * −33 (13) −30 (8) * −26 (4) −32 (9)	/ / / 22 (0) 29 (7) /	Yes	High

* The results are all simulated results. N.A.: Not available.

## Data Availability

The data presented in this study are available on request from the corresponding author.
